# Molecular community profiling of the bacterial microbiota associated with denture-related stomatitis

**DOI:** 10.1038/s41598-019-46494-0

**Published:** 2019-07-15

**Authors:** Daniel J. Morse, Ann Smith, Melanie J. Wilson, Lucy Marsh, Lewis White, Raquel Posso, David J. Bradshaw, Xiaoqing Wei, Michael A. O. Lewis, David W. Williams

**Affiliations:** 1 Microbiomes, Microbes and Informatics Group, School of Biosciences, Cardiff University, Cardiff, UK; 2 Oral and Biomedical Sciences, School of Dentistry, Cardiff University, Cardiff, UK; 3 School of Medicine, Cardiff University, Cardiff, UK; 4 UKCMN Regional Mycology Reference Laboratory, Public Health Wales, Microbiology Cardiff, Cardiff, UK; 5 GlaxoSmithKline Consumer Healthcare, Weybridge, UK

**Keywords:** Clinical microbiology, Clinical microbiology, Microbiome, Epidemiology

## Abstract

Denture-associated stomatitis (DS) affects over two-thirds of denture-wearers. DS presents as erythema of the palatal mucosa in areas where denture-surface associated polymicrobial biofilms containing the fungus *Candida albicans* exist. The contribution of the oral bacterial microbiota toward the infection is unknown. Therefore, this study characterised the bacterial microbiota of sites within the oral cavity to identify potential associations with occurrence of DS. Denture-wearing patients were recruited (denture stomatitis (DS) n = 8; non-denture stomatitis (NoDS) n = 11) and the oral bacterial microbiota of the tongue, palate and denture-fitting surface was characterised using next-generation sequencing. Operational taxonomic units (OTUs) were identified to bacterial genera and species, and presence/absence and relative abundances were examined. A significant (*P* = 0.007) decrease in the number of OTUs and thus, diversity of the microbiota was observed in tongue samples of DS patients (vs non-DS). The microbiota of denture-fitting surfaces and palatal mucosae were similar. Large differences in the abundance of bacterial genera and species were observed at each sample site, and unique presence/absence of bacteria was noted. Presence/absence and relative abundance of specific bacteria associated with DS warrants further *in vitro* and *in vivo* evaluation, particularly as our previous work has shown *C*. *albicans* virulence factor modulation by oral bacteria.

## Introduction

The microbiota of higher organisms, and particularly humans, are of great interest given their affiliation with homeostasis of the host and normal function of many different body sites. There is substantial interest in the onset of disease that may be attributed to the endogenous microbiota, fuelled by the view that whilst these communities exist naturally at sites in the human body without negative impact toward the host, changes in the composition of the microbiota can lead to dysbiosis and development of localised and systemic infection.

Microbial communities are crucial to the normal function of many different environments in the human body, such as the gastrointestinal tract^1,2^ where microbial communities contribute to food breakdown, providing nutrients to surrounding host cells, thus developing a mutually beneficial relationship between the host and the resident microbiota. However, this benefit is not always maintained. Factors including the use of antibiotics, or significant changes in diet can lead to altered proportions of microorganisms in the gut microbiota, leading to dysbiosis and potential infection^3–5^.

Dysbiosis also applies to the oral cavity, where even subtle changes can lead to disease. This is evident in dental caries and periodontal disease, where an increase in the proportion of known ‘keystone’ pathogens, including *Streptococcus mutans* or *Porphyromonas gingivalis*, leads to substantial destruction of enamel and surrounding tissue, respectively^6–10^.

Various microenvironments exist within the oral cavity, offering a number of unique surfaces for microbial colonisation *e*.*g*. hard tooth enamel surfaces and soft tissues. Each location supports a distinct microbiota, arising from, among other factors, the diverse environmental conditions, physical environment and nutrient availability. The resident oral microbiota can inhibit the development of diseases including dental caries^11^ by minimising levels of potentially pathogenic microorganisms^10,12^, through direct competition for colonisation and/or nutrients^13^.

Several studies have characterised the healthy bacterial microbiota at different sites in the oral cavity, including tooth surfaces, oral mucosae and saliva^9,12,14^, and The Human Oral Microbiome Database (HOMD), established in 2010, indicates that over 700 bacterial species exist in the mouth^15^.

In addition to bacteria within the oral microbiota, fungi play an increasingly acknowledged role in the onset of disease. Oral candidosis, for example, is one such frequently occurring, but somewhat underappreciated infection. *Candida albicans* remains the most frequent *Candida* species isolated from the oral cavity and is widely regarded as the primary cause of chronic erythematous candidosis (also known as denture-associated stomatitis; DS). DS affects approximately 67% of denture wearers^16^, presenting as erythema of the palatal mucosa in areas that are in direct contact with the fitting-surface of a complete or partial denture. The extent of inflammation can be categorised according to Newton’s classification^17^: Type 0, no erythema; Type 1, localised, pinpoint erythema; Type 2, diffuse erythema (moderately red, covering part or all of the area of denture contact); Type 3, hyperplastic granular inflammation (severely red or swollen palatal mucosa)^18–20^.

Although it is recognised that *C*. *albicans* is the primary causative microorganism of DS, several predisposing factors known to promote the condition have been documented including tobacco use, nocturnal denture wearing, ill-fitting dentures, and poor oral and denture hygiene. However, our knowledge of the role of the bacterial microbiota and the contribution toward DS is poor.

*C*. *albicans* is described as an opportunistic pathogen, with the ability to exist in a commensal yeast-like form, and can undergo a morphogenic transition to elongated hyphae, considered the pathogenic form^21^. Previously, we have shown that biofilms containing oral bacteria and *C*. *albicans* result in significantly greater tissue damage and an increased immune response relative to *C*. *albicans*-only biofilms^22,23^. Furthermore, in these previous studies, *C*. *albicans* hyphae, and the expression of a range of virulence factors including hydrolytic enzymes, were shown to increase when *C*. *albicans* was cultured with oral bacteria in mixed-species biofilms. We have also found that the inclusion of other oral bacteria, albeit another keystone pathogen (*Porphyromonas gingivalis*), in mixed-species biofilms can further modulate the virulence of *C*. *albicans*, leading to reduced *C*. *albicans* virulence^24^.

As a result of these *in vitro* findings, the aim of this study was to characterise the bacterial microbiota at three oral sites (tongue, hard palate, and fitting-surface of the denture) in patients with and without clinical signs of DS, to further our understanding of the potential involvement of oral bacteria in DS.

## Results

### Patient demographics

A total of 19 patients were recruited to the study (denture stomatitis (DS) n = 8; non-denture stomatitis (NoDS) n = 11). The demographic and clinical data for the subjects are presented in Table 1. Samples were assigned an anonymised reference number (S001-S019). The sample ID included reference to the sample site; where T referred to tongue, P to palatal mucosa, and D was the denture-fitting surface (*e*.*g*. S001T, patient 1 tongue sample site).

**Table 1:** Patient demographics recorded at recruitment for each individual patient (anonymised).

Patient ID	Gender	Age (yrs)	Tobacco smoker	Denture stomatitis	
				Present	Newton’s Classification
S001	Female	59	Yes	No	0
S002	Female	66	No	Yes	1
S003	Male	66	No	No	0
S004	Male	66	No	No	0
S005	Male	52	Yes	No	0
S006	Male	64	No	No	0
S007	Female	67	No	Yes	1
S008	Female	75	No	No	0
S009	Male	72	Yes	No	0
S010	Female	74	Yes	Yes	2
S011	Female	36	Yes	Yes	1
S012	Female	81	No	No	0
S013	Male	75	Yes	Yes	3
S014	Male	65	Yes	Yes	2
S015	Female	72	No	No	0
S016	Female	90	No	Yes	2
S017	Male	59	No	No	0
S018	Female	77	No	No	0
S019	Female	69	Yes	Yes	2

More females (n = 11) were recruited than males (n = 8), and similar average ages 69.64 years (±13.84) and 64.88 years (±7.14) were evident for both genders, respectively. Of the recruited patients, eight (42.1%) were current smokers.

Eight patients had DS to varying extent (Female n = 6; Male n = 2), whereas 11 individuals had no signs of DS (Female n = 5; Male n = 6).

### Detection of *Candida* species by culture and PCR

Detection of *Candida* is detailed in Table 2. Patients that were positive for *Candida* by either agar culture or nested PCR were included in the bacterial community profiling analysis.

**Table 2:** *Candida* presence and presumptive identification by culture and PCR.

Patient ID	*Candida* presence			Presumptive *Candida* species identification (CHROMagar/RT-PCR)
	Tongue (Culture/PCR)	Palate (Culture/PCR)	Denture (Culture/PCR)	
S001	+/NT	+/NT	+/NT	*C*. *albicans*
S002	−/NT	−/NT	−/NT	N/A
S003	−/−	−/−	−/+	*Candida* spp.
S004	+/+	+/+	−/+	*C*. *albicans*
S005	+/+	+/+	−/−	*C*. *albicans*
S006	−/−	−/+	−/+	*Candida* spp.
S007	−/+	−/−	−/+	*Candida* spp.
S008	−/+	−/+	−/+	*Candida* spp.
S009	−/+	+/NT	−/+	*C*. *albicans*
S010	−/+	−/+	+/NT	*C*. *albicans*
S011	+/+	+/NT	+/+	*C*. *albicans*
S012	+/+	+/NT	+/+	*C*. *albicans*
S013	−/−	−/−	−/+	*Candida* spp.
S014	+/−	−/+	+/NT	*C*. *albicans*
S015	−/−	−/−	−/+	*C*. *albicans*
S016	−/−	−/−	−/−	N/A
S017	−/−	−/−	−/−	N/A
S018	−/−	−/−	−/−	N/A
S019	+/NT	+/NT	+/NT	*C*. *albicans*

*Candida* presence (+) or absence (−) determined by positive agar culture and real-time PCR identification. Samples not tested by real-time PCR are denoted by NT. Samples that were positive by culture were further subcultured onto CHROMagar® *Candida* for presumptive identification of *Candida* species. N/A represents samples negative for detection of *Candida* by both culture and molecular analyses.

Several samples that did not yield *Candida* growth by agar culture did test positive for *Candida* by the nested PCR method. These samples were S003D, S006P, S006D, S007T, S007D, S008T, S008P, S013D, S014P, S015D. In these sample descriptions, D, P and T represent denture, palate and tongue, respectively. Conversely, *Candida* was not detected by molecular testing of the S014T sample but was detected by culture.

From each sample type (*i*.*e*. tongue, palate or denture) on SDA, several typical colonies were subcultured on to CHROMagar® *Candida* agar. The agar plates were incubated for 48 h, after which, a turquoise/green colour was observed for colonies, indicating presumptive identification of *C*. *albicans*.

Nested real-time PCR was used to identify *Candida* in samples to species level. Amplicons were observed in the pan-*Candida* channel for numerous samples, but no detection was evident in the *C*. *glabrata* or *C*. *krusei* probe channels, thus eliminating the presence of these species. These results, when considered alongside those of culture, indicated the presence of *Candida*, presumably *C*. *albicans*, in all tested samples, and subsequent germ tube tests confirmed the ability of isolates  to form hyphae in the presence of serum.

### Metataxonomic profiling of bacterial species present in swab samples

Of the 57 samples collected, 55 were processed for DNA sequencing. Five failed to yield amplicons in the primary stage, thus the remaining 50 samples were used in subsequent analyses. A total of 2,194,967 sequence reads were obtained, and after quality control steps, the total number of final reads was 1,864,575. To normalise between samples, the OTUs were sub-sampled to the lowest read count (S019P) of 10,995 reads.

Patients were grouped by disease status *i*.*e*. presence or absence of clinical symptoms of DS (DS or NoDS, respectively), and analysed. In total, 2,411 OTUs were identified, 353 of which had more than ten sequence reads. Bacterial genera and species with ten or more sequence reads were included in analysis.

Duplicate bacterial species (which can arise due to strain sequence variation) when phylotypically classified were merged, along with their OTU and relative proportion data. Thus, the overall number of unique bacterial species was lower than the number of OTUs detected. Both were considered independently for the purpose of this study.

### Differences in bacterial microbiota of DS and Non-DS patients

**Figure 1:**
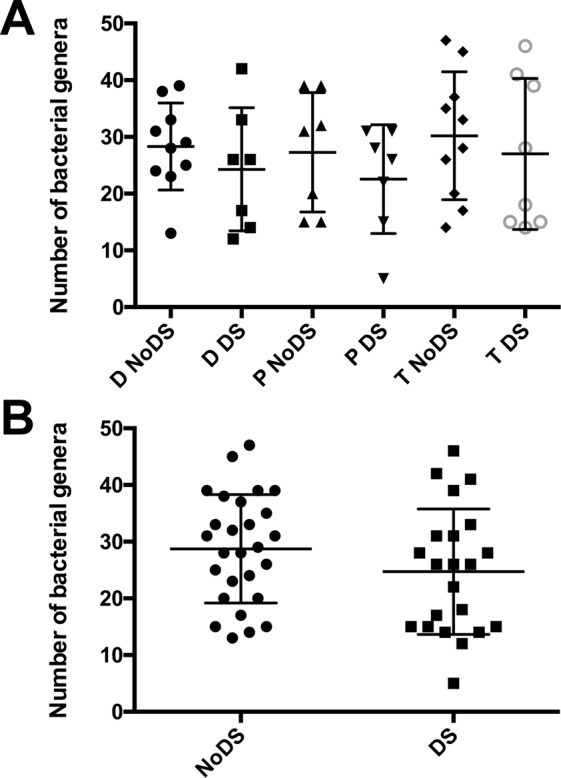
Scatter plot of number of unique bacterial genera grouped by (**A**) sample site and DS state, and (**B**) DS state only. The number of bacterial genera for each patient when grouped by both sample site and DS state, and by DS state only, showing spread and error bars represent standard deviation. Similar mean numbers of bacterial genera were evident across all groupings, with no statistically significant differences.

Quantities and relative proportions of bacterial genera were compared within sample sites (e.g. DS versus NoDS in samples of the tongue, palate and denture-fitting surface), and between sample sites, *e.g.* (Tongue DS versus Palate DS versus Denture DS). No significant (*P* > 0.05) differences were evident when comparing within or between sample sites for the number of genera when considering patients grouped by disease state and sample site (Fig. 1A). Similarly, no significant (*P* = 0.191) differences were evident when analysing patients grouped by disease status only (Fig. 1B).

**Figure 2:**
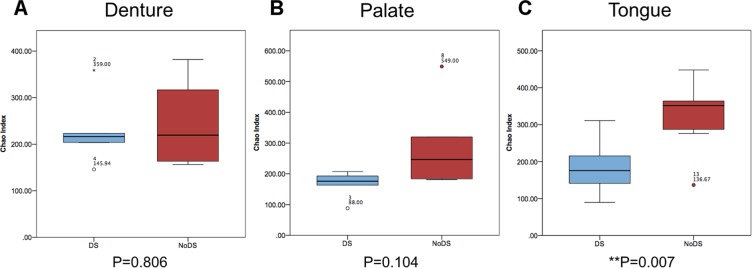
Box and whisker plot showing Chao index analysis for each sample site. Number of unique bacterial operational taxonomic units (OTUs) for patients with DS (blue) compared with patients without DS (red) in samples of the (**A**) denture-fitting surface, (**B**) palatal mucosa surface and (**C**) tongue. A significant (*P* = 0.007) reduction in the number of detected OTUs was observed in samples from the tongue of DS patients, but strong similarities in samples of the denture-fitting surface and the palate were observed.

The number of unique OTUs was determined for each sample (Chao index) (Fig. 2). The number of unique OTUs in samples of the denture-fitting surface (Fig. 2A) showed no significant differences (*P* = 0.806) between DS and NoDS patients. Similarly, samples from the palatal mucosa (Fig. 2B) in the same patients showed a slight increase in the number of unique bacterial species for NoDS patients, but this was not statistically significant (*P* = 0.104). However, a significant (*P* = 0.007) increase in the number of unique bacterial species was observed in samples from the tongue (Fig. 2C) of NoDS compared with DS patients.

**Figure 3:**
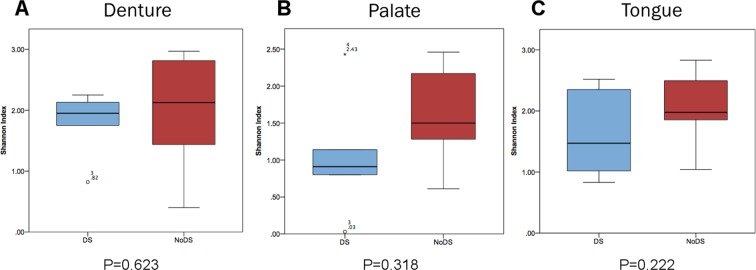
Box and whisker plot showing Shannon index analysis for each sample site. The richness and evenness of spread related to abundance of bacterial operational taxonomic units (OTUs) from patients with DS (blue) compared with patients without DS (red) in samples of the (**A**) denture-fitting surface, (**B**) palatal mucosa surface and (**C**) tongue. A general increase in Shannon index of each sample site indicated higher diversity and more equal distribution of abundance in samples from patients without clinical presentation of DS compared with patients with DS.

The Shannon index was calculated (Fig. 3) as an indication of OTU abundance and evenness of spread, to indicate diversity within the samples. No significant differences (*P* > 0.05) were observed between DS and NoDS patients for any sample site. This was indicative that the frequency of OTUs was more evenly distributed across the whole population, rather than clusters of higher relative abundance associated with a smaller group of specific bacterial species. A slightly higher Shannon index was observed for NoDS samples from the palatal mucosa than DS samples, indicating that differences in the evenness of distribution between the frequency of bacterial species may have been present, but the differences were subtle and not significant.

**Figure 4:**
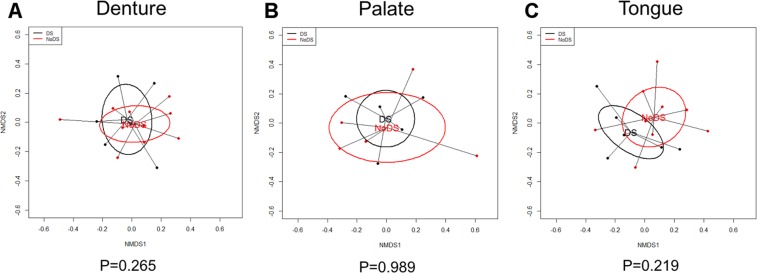
Nonmetric multidimensional scaling (NMDS) plots of denture-associated stomatitis (DS) versus non-DS (NoDS) groups. NMDS ordination technique analysis of samples within DS and NoDS groups for each sample site to identify distinct clusters of groupings. (**A**) Denture-fitting surface, (**B**) palatal mucosa surface and (**C**) tongue. Overlapping groupings of samples were observed, thus the overall bacterial microbiota were not distinct.

Communities of microorganisms can be grouped by overall similarities of the bacterial microbiota and presented graphically to show whether or not the clusters are distinct. Where clusters of samples overlap, they are deemed not dissimilar. Non-metric multidimensional scaling plots were generated for each sample site (Fig. 4). The spread of the data points and the trends indicated by the ellipses of the patient groupings of DS and NoDS patients overlapped considerably in this analysis for all sample sites. This indicated the bacterial microbiota as a whole were not considered distinct, but as such showed greater overall similarities between the healthy and disease states. The spread within the results indicated some variation between samples, and inherent variability between patients.

### Presence and differences in relative abundance of bacteria analysed at genera and species level

**Figure 5:**
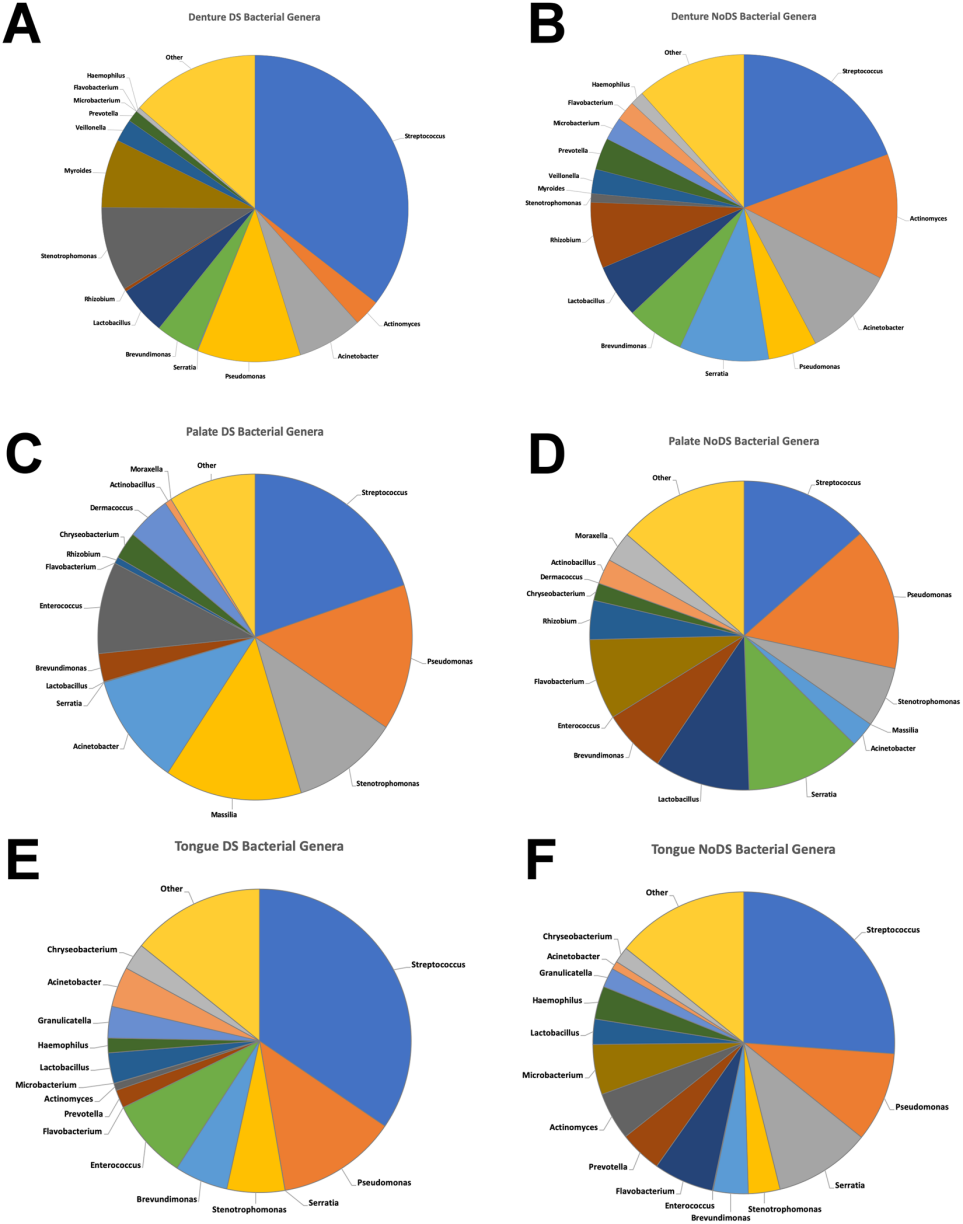
Pie chart graphical representations showing mean relative abundances of bacterial genera at each sample site. Top 15 bacterial genera (by mean abundances) in samples of the denture-fitting surface from patients (**A**) with DS and (**B**) without DS, samples of the palate from patients (**C**) with DS and (**D**) without DS, and samples of the tongue from patients (**E**) with DS and (**F**) without DS.

Despite no differences in the genera-associated microbiota being evident when considered collectively (Fig. 5), when analysing relative abundance differences between DS and NoDS at each sample site, some striking differences were detected. Data presented in Fig. 5 (with corresponding specific numerical proportions presented in Supplementary Tables S1–S3) demonstrate the proportion of each detected genera as a mean value of all the participants in that sample group, where many substantial differences were observed. In samples of the denture-fitting surface (Fig. 5A,B), a number of shifts were observed, particularly in the genera associated with the top 15 genera by mean abundance. In DS-associated samples, increases in the relative abundance of *Streptococcus*, *Pseudomonas*, and *Stenotrophomonas* were evident, with decreases in the relative abundance of *Actinomyces*, *Serratia*, and *Rhizobium*.

Shifts in relative abundances of genera were also observed in samples of other sites, including large increases in relative abundances observed in *Streptococcus*, *Masillia* and *Acinetobacter* in samples of the palate (Fig. 5C,D), and *Streptococcus* and *Enterococcus* in samples of the tongue (Fig. 5E,F). Large decreases in genera including *Serratia*, *Lactobacillus* and *Flavobacterium* were evident in samples of the palate, and similarly, decreases in *Serratia* and *Flavobacterium* were observed in samples of the tongue.

**Figure 6:**
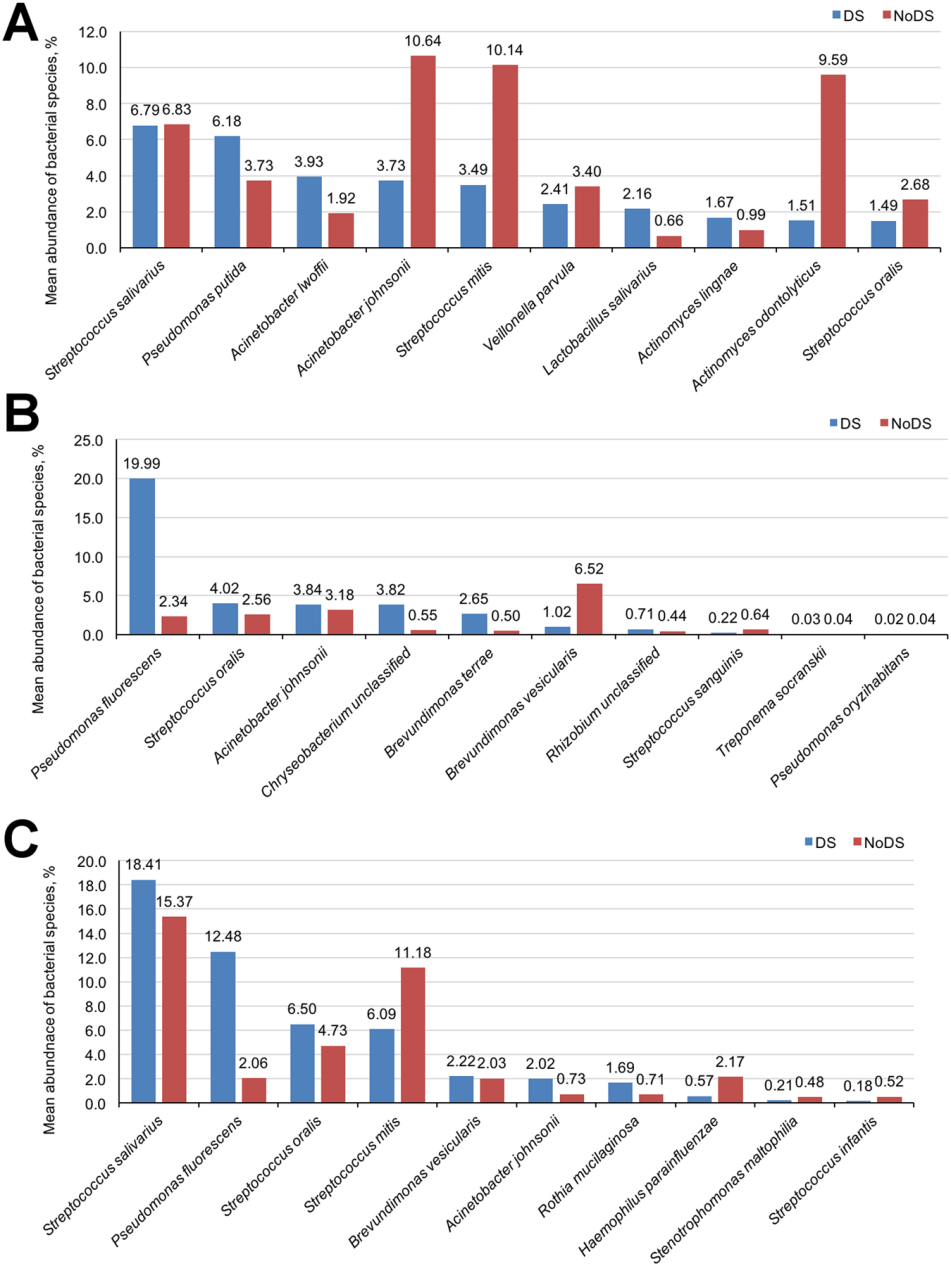
Average relative abundance of top 10 individual bacterial species common to both DS (blue) and NoDS (red) patient groups in samples of the (**A**) denture-fitting surface, (**B**) palate, and (**C**) tongue.

When considering the microbiota at species level, the relative proportion of different bacterial species varied considerably between samples and patients (Fig. 6; Supplementary Tables S4–6). Overall, the top 25 bacterial species by average abundance accounted for more than 85% of the total abundance of bacterial species, and in the case of samples from the palatal mucosa of DS patients, accounted for 98% of the overall abundance.

Hundreds of unique bacterial species were detected in each of the samples. Many species were unique to either DS or NoDS patients and may contribute to the incidence of DS. However, of the bacterial species found in both DS and NoDS patients, some substantial differences in the relative abundance were detected (Fig. 6).

In samples of the denture-fitting surfaces of NoDS patients (Fig. 6A), the relative proportions of *Acinetobacter johnsonii* and *Streptococcus mitis* were nearly three-fold higher compared with DS patients, and proportions of *Actinomyces odontolyticus* were nearly six-fold higher. Additional differences were also observed, but to a lesser extent. These included a reduction in the presence of *Pseudomonas putida*, *A*. *lwoffii*, *Lactobacillus salivarius* and a slight decrease in *A*. *lingnae* in samples from NoDS patients. Furthermore, two of the three most abundant bacterial species in samples of the fitting surface of the denture were unique to the DS group. These included an unknown species within the genus *Myroides*, and *Pseudomonas fluorescens*, with relative abundances of approximately 8.5% and 7.2%, respectively. The presence of *Myroides* was not detected at any other sample site.

Further differences were observed for microbiota of the palatal mucosa (Fig. 6B). *Pseudomonas fluorescens* was the bacterial species with highest representation in DS patients, but was also detected in NoDS patients. There was, however, a substantial difference in the average relative abundance, with a decrease from 19.99% in DS patients to 2.34% in NoDS patients. This was the largest difference for all bacterial species, irrespective of sample site. Furthermore, a greater than six-fold increase in the proportion of *Brevundimonas vesicularis* in NoDS patients compared with DS patients was evident. Conversely, a reduction of *S*. *oralis*, *Chrysobacterium* spp., *B*. *terrae* and a slight decrease in *A*. *johnsonii* were observed in NoDS patients. As was also evident in samples of the fitting surface of dentures, a number of unique bacterial species were detected in the DS and NoDS groups.

*Streptococcus salivarius* was detected at the highest relative abundance from tongue samples (Fig. 6C). A small reduction was observed between DS and NoDS patients of approximately 3% (DS = 18.41%, NoDS = 15.37%), but the abundances were still higher than all other bacteria detected in either the tongue samples of DS or NoDS patients. Large differences were observed in the proportion of *P*. *fluorescens* between DS (12.48%) and NoDS (2.06%), and *S*. *mitis* (DS = 6.09%, NoDS = 11.18%). Smaller differences were observed with other bacterial species common between DS and NoDS patients, but their proportional contribution to the microbiota was approximately 2% or less, thus the extent of the differences were less clear.

**Figure 7:**
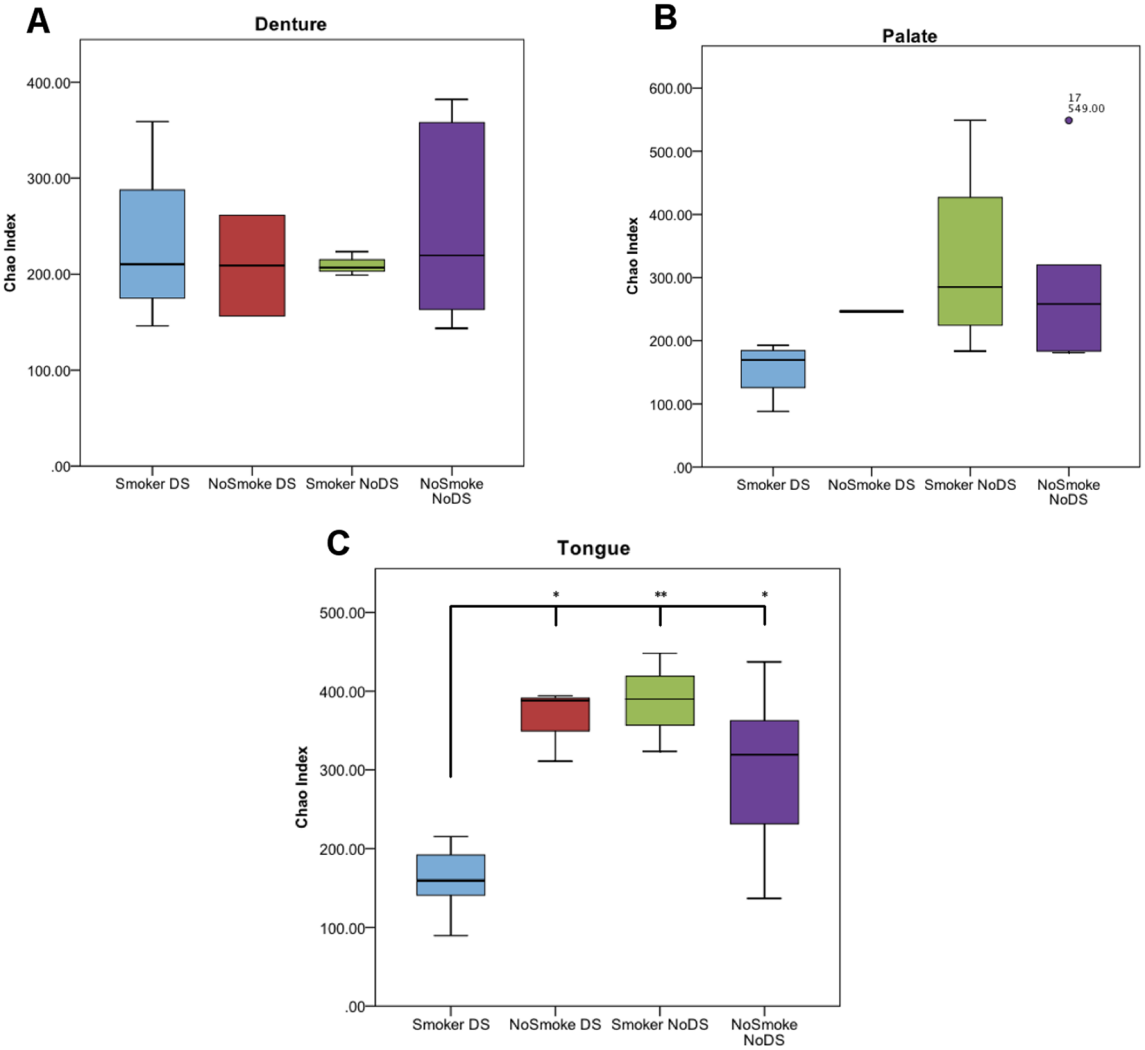
Box and whisker lot showing Chao index analysis, with samples grouped by sample site, DS state and sub-grouped by smoking status. A significant reduction was observed in smokers with DS relative to non-smokers with DS and non-smokers without DS (*P* < 0.05) and smokers without DS (*P* < 0.01) in samples of the tongue, but no significant differences were observed in samples of the denture-fitting surface. Samples of the palate showed a similar trend to that of the tongue, but with no statistical significance.

When patients were sub-grouped by smoking status, some clear differences in OTU relative abundances were evident. Strong similarities in the numbers of OTUs detected from samples of the denture-fitting surface were evident between smokers and non-smokers, irrespective of DS state (Fig. 7A). In samples from biotic sites (Fig. 7B,C), however, smokers with DS had a much reduced quantity of OTUs detected than all other groups, significantly (*P* < 0.05 vs non-smokers with DS and non-smokers without DS, and *P* < 0.01 versus smokers with DS) so in samples from the tongue (Fig. 7C).

## Discussion

This research represents the first clinical study using next generation sequencing to characterise denture-associated biofilms from patients with and without clinical presentation of DS, with a specific aim of characterisation to species level to associate presence/absence and abundance with incidence of disease. Previous studies have evaluated DS associated microbiota, but the data were only analysed to genus level^25,26^. Campos *et al*., (2008) completed a similar study to the one reported here, using pooled swab samples of DS versus NoDS patient groups, but also only reported data at genus level^27^.

Genus-level information is important, but it is also well documented that considerable differences exist between species of the same genera. In the oral context, *S*. *salivarius* and *S*. *mutans* are good examples of distinct species within the same genus, particularly in terms of virulence and pathogenicity. *Streptococcus salivarius* is considered a commensal bacterial species, not normally associated with oral infection, whereas *S*. *mutans* is a known pathogen, associated with the development of dental caries. Furthermore, a study by Kim *et al*., (2017) demonstrated inter-kingdom interactions between *S*. *mutans* and *C*. *albicans*, where the presence of *C*. *albicans* resulted in an increase in the formation of microcolonies and subsequent virulence of *S*. *mutans*^28^. Therefore, an increase in proportion and virulence of a particular bacterial species may lead to a worse overall prognosis, and needs to be considered within the wider context of the microbiota.

NGS data did not show any significant (*P* > 0.05) differences in the overall bacterial microbiota between patients with or without DS for the denture-fitting surface, or palatal mucosa. Indeed, the results for these samples were similar for both the number of unique species detected (Chao index), and in the richness and evenness of spread (Shannon index). However, in samples from the tongue, a significant (*P* = 0.007) decrease was observed in the number of unique bacteria species in patients with DS, compared to those without DS. This is interesting because a reduction in biodiversity, resulting in a state of dysbiosis, can lead to development of more extreme local environments where microorganisms with more extreme tolerances can thrive. Such changes in environmental conditions have been observed in the lung and the gut, and as a result of these changes, the onset of dysbiosis occurs, leading to an exacerbation of susceptibility to infections and a reduction in function particularly in the strictly-managed environment of the lung^29,30^. Subsequently, the behaviour of these microorganisms, and the way they utilise nutrients and contribute to the local environmental conditions can further damage the local environment, such as the host^31,32^.

When analysing individual bacterial species, some clear and clinically interesting differences were evident. The abundance of individual bacterial species within sample groups revealed some notable differences. For example, in samples from the denture-fitting surface, a bacterial species within the genus *Myroides* (species unknown) was found exclusively in the DS sample group (at approximately 8.5% relative abundance). *Myroides* (specifically the species *M*. *odoratimimus*) have been implicated in soft tissue infections of immunocompetent patients^33^, and linked with cases of bacteraemia in diabetic patients^34^, which is also a risk-factor for DS. It is therefore possible that this microorganism in the DS group could contribute to palatal inflammation. Alternatively, the presence of bacteria could also contribute to the local microbial community (biofilms) residing on the denture surface, and the modulation of *Candida* virulence, thus indirectly promoting palatal inflammation.

*Pseudomonas fluorescens* was detected exclusively in the denture samples from DS patients with an abundance of approximately 7%. This species was however also detected in both DS and NoDS samples of the palate and tongue, indicating its presence was not necessarily by chance, but it had successfully colonised each of these sites. There were notable differences in the abundance of *P*. *fluorescens* between DS and NoDS from palate (DS = 20%, NoDS = 2.3%) and tongue (DS = 12.5%, NoDS = 2%) samples. At both biotic sites (palate and tongue), there was therefore a basal level of *P*. *fluorescens* presence of approximately 2% of the overall bacterial microbiota. It is unclear whether the change in the abundance was caused by DS, or that it was responsible for the onset of DS. DS is a very complex infection with many influencing factors, where no apparent single factor contributes solely to the incidence or development of the disease.

In anticipation of future clinical studies, it would be of interest to obtain additional clinical information of each patient, beyond the smoking status and demographic details. Information such as presence of diabetes, HIV status, diet, previous episodes of oral candidosis, denture state (*e.g.* age of denture, physical condition, cleaning regime), and dental health (including number of remaining teeth and oral hygiene regime) could have been assessed. Each of these may influence the conditions in the mouth, and thus, the resident microbiota. Poor glycaemic control due to diabetes has been associated with increased susceptibility to DS, as have HIV infection, smoking and poor oral hygiene, and with this limited sample set, although it would have been difficult to make statistically valid associations, an indication of trends may have provided foundation for additional studies to evaluate these parameters.

The sample collection, extraction and utilisation of DNA provided detailed information of what bacteria were present, but not necessarily whether they were viable or metabolically active. This is an interesting area to consider, as the mere physical presence of microorganisms, irrespective of viability can elicit an immune response to the physical cell surface  components such as mannans and β-d-(1,3)-glucans of *Candida*^35,36^, and lipopolysaccharide or peptidoglycan of Gram-negative and Gram-positive bacterial cells respectively^36,37^. Viable bacteria, particularly pathogens, will arguably lead to a worse prognosis without intervention, but follow up studies would be required, and it would be of scientific value to establish a baseline microbiota, then monitor changes with incidence of disease.

## Conclusions

Although differences in the overall bacterial microbiota between sample sites were evident, considerable differences between DS and non-DS patients were observed for microbiota of the tongue. The tongue is an important reservoir of microorganisms for the oral cavity and the oropharynx, and is known to harbour many anaerobic microorganisms. The reduction in biodiversity demonstrated in patients with DS suggested that dysbiosis may contribute to the onset of disease/infection, whereby the relative influence of bacterial species of higher abundance within the community is further enhanced. These findings, taken together with our previous research documenting the bacterial modulation of *Candida* virulence, support the need for further exploration of the bacterial microbiota associated with DS and the importance of characterisation of complex communities to species level.

## Materials and Methods

### Ethical approval

Research ethics committee approval was obtained (Wales REC 1) with the following reference information: Study title: Denture acrylic biofilms: microbial composition, interactions and prevention; *REC reference*: 14/WA/0023*; Protocol number*: SPON 1265-13*; IRAS project ID*: 137108. Patients attending the University Dental Hospital, Cardiff and Vale University Health Board, for routine treatment were recruited following informed consent.

### Patient recruitment and sample collection

In order to achieve a robust consistency between recruited individuals, strict criteria were used to exclude factors that are known to substantially impact the oral microbiome. Patients were required to be over the age of 18 years, able and willing to consent, and have a complete upper acrylic denture. Patients who had received antibiotics or antifungal drugs, used steroids (systemic or inhaler), immunosuppressant or investigational drugs, or participated in another clinical study in the 30 days prior to recruitment, were not eligible for recruitment to the study.

The clinical information recorded for each patient included: gender, age, smoking status, and the presence and extent of denture stomatitis according to Newton’s classification.

### Clinical sampling

Samples were obtained by clinically qualified and trained dental professionals employed by the Cardiff University School of Dentistry. The dental professionals worked under the guidelines and regulation of the General Dental Council, and sampling performed according to best practices.

Prior to any clinical intervention, the fitting-surface of the denture, the tongue and the hard palate were sampled using separate sterile Transwab® Amies Charcoal swabs (Medical Wire and Equipment, Wiltshire, UK), where the swabs were rubbed across each site for 15 s.

Additionally, for detection and isolation of *Candida*, individual 2 cm^2^ sterile foam squares pre-soaked in phosphate buffered saline solution (PBS) were pressed against the denture fitting surface, tongue and hard palate for 30 s. The squares were then placed directly onto a Sabouraud dextrose agar (SDA) plate in the clinic prior to immediate transfer to the laboratory for aerobic incubation for 24–48 h at 37 °C. Random representative colonies considered typical for *Candida* were subcultured onto CHROMagar® *Candida* (BD Biosciences, Oxford, UK), and incubated for a further 48 h.

### Laboratory processing of swab samples

Prior to collection of clinical samples, validation of the sampling process involving testing charcoal swabs was performed. Swabs of *in vitro* biofilms cultured using *Streptococcus sanguinis* NCTC 7863 and *Candida albicans* ATCC 90028 were collected, and the DNA extracted using the ‘Gram-positive bacteria’ extraction protocol of the Gentra PureGene Bact/Yeast DNA extraction kit (Qiagen, USA) as detailed by the manufacturer. The extracted and purified DNA was then subjected to endpoint PCR amplification. Positive amplification was observed confirming the suitability of the method. The relative level of microorganisms within clinical samples was anticipated to be less than would be observed in *in vitro* biofilms, therefore nested-PCR for detection of *Candida* species was incorporated to enhance sensitivity of detection.

Under aseptic conditions, the swab tip was carefully separated from the stem and placed into a sterile bijou container containing 1 ml of PBS, then vortex mixed at 2,500 rev/min for 1 min. The supernatant was collected, thoroughly mixed and transferred to a sterile microcentrifuge tube and centrifuged for 2 min at 13,000 × *g*. The supernatant was discarded and 300 μL of sterile PBS added. The suspension was then homogenised by pipetting. Aliquots of the suspension were prepared for DNA extraction for bacterial and fungal molecular analyses.

### Total microbial DNA extraction from clinical swab samples

Total bacterial DNA was extracted using the Gentra PureGene Bact/Yeast DNA extraction kit (Qiagen, USA) according to the Gram-positive bacteria extraction protocol provided by the manufacturer. Extracted DNA was stored at −20 °C prior to next generation sequencing.

In addition to culture, *Candida* presence was established by nested PCR. A portion of the swab sample was used for extraction of yeast DNA using the Gentra PureGene Bacteria/Yeast DNA extraction kit, according to the yeast/fungi extraction protocol.

### Identification of *Candida* from clinical samples by nested PCR

Extracted fungal DNA was pre-amplified using a general nested PCR and the RenDX Fungiplex Amplification Kit (Renishaw Diagnostics Ltd, Glasgow, UK) in a final reaction volume of 50 μL. The method targets the region spanning positions 620–760 on the fungal 18S rRNA gene. Each reaction mix contained 5 μL of 10 × PCR buffer, 1 μL MgCl_2_, 4 μL dNTPs, 10 μL primer mix (final concentration 600 mM), 19.5 μL molecular grade water, 0.5 μL (2.5 U) *Taq* polymerase and 10 μL of the extracted DNA template. Target DNA was amplified using the following thermal cycling protocol: 95 °C for 15 min, followed by 45 cycles of 94 °C for 30 s, 58 °C for 30 s and 72 °C for 30 s. A final elongation step of 72 °C for 7 min was performed. Samples were held at 4 °C until required for the next stage of amplification.

Amplified samples were subjected to a second round of probe-based real-time PCR to amplify 18S ribosomal RNA (18S rRNA) sequences specific to pan *Candida* species, *C*. *krusei* and *C*. *glabrata*. The primers used are detailed in Supplementary Table S7.

DNA samples were amplified in the second round using LightCycler FastStart DNA Master HybProbe (Roche Diagnostics, Sussex, UK), and reactions contained 2.5 μL of hybridisation mix, 3 μL of MgCl_2_, 15 μL of nuclease free molecular biology grade water, 2.5 μL of primer/probe mix and 2 μL of template DNA. The preparations were homogenised by brief vortex mixing. DNA was amplified using the following thermal cycling protocol: 95 °C for 15 min, followed by 30 cycles of 95 °C for 15 s, then 58 °C for 30 s. Amplicons were detected using the following fluorescence probes FAM (green), JOE (yellow) and ROX (orange) for detection of pan-*Candida* species, *C*. *glabrata* and *C*. *krusei*, respectively.

### Metataxonomic profiling of bacterial microbiota of clinical swab samples using the Illumina MiSeq two stage amplification protocol

Bacterial DNA was sequenced by Research and Testing Laboratories (RTL, Texas, USA) using an Illumina 2-step protocol as outlined below.

### Primary stage amplification

The forward primer was constructed (5′-3′) with the Illumina i5 sequencing primer (TCGTCGGCAGCGTCAGATGTGTATAAGAGACAG) and the 28F primer (GAGTTTGATCNTGGCTCAG), and the reverse primer was constructed with (5′-3′) the Illumina i7 sequencing primer (GTCTCGTGGGCTCGGAGATGTGTATAAGAGACAG) and the 388R primer (TGCTGCCTCCCGTAGGAGT). This region spans the V1-V3 hypervariable regions of the bacterial 16S rRNA gene. Reactions were performed using ABI Veriti thermocyclers (Applied Biosystems, California, USA) in a 25 μL final volume comprising 22 μL of Qiagen HotStar *Taq* master mix (Qiagen Inc. California, USA), 1 μL of each primer (5 μM) and 1 μL DNA template. The thermal cycling protocol was: 95 °C for 5 min, then 25 cycles of 94 °C for 30 s, 54 °C for 40 s, 72 °C for 1 min, followed by one final cycle of 72 °C for 10 min; the products were then held at 4 °C.

### Second stage amplification

Products from the first stage amplification were added to a second PCR, based on qualitatively determined concentrations. Primers for the second PCR were designed based on the Illumina Nextera PCR primers and were as follows: Forward - AATGATACGGCGACCACCGAGATCTACAC[i5index]TCGTCGGCAGCGTC and Reverse - CAAGCAGAAGACGGCATACGAGAT[i7index]GTCTCGTGGGCTCGG. The thermal cycling protocol for this second stage amplification was the same as the first but limited to 10 cycles.

### Standardisation of PCR products for next-generation sequencing

Amplicons were initially visualised using eGels (Life Technologies, New York, USA). Products were pooled at equimolar concentrations and each pool was size selected in two rounds using Agencourt AMPure XP (BeckmanCoulter, Indiana, USA) in a 0.7 ratio for both rounds. Size selected pools were then quantified using the Qubit 2.0 fluorimeter (Life Technologies) and loaded on an Illumina MiSeq (Illumina, Inc., California, USA) 2 × 300 flow cell at 10 pM.

### Data analysis

The 16S rRNA gene sequences generated were analysed using the open source bioinformatics software package, Mothur v 1.36.1^38,39^ using the MiSeq SOP Pipeline to analyse 50 samples. The sequence alignment was performed using the Silva bacterial database^40^ as the reference, and classification of sequences were undertaken using the RDP (Ribosomal Database Project) database reference sequence files and the Wang method^41^. The OTU taxonomies (phylum to genus) were determined using the RDP Classifier script, and at species-level taxonomies was determined using USEARCH^42^ at ≥97% similarity. The cohorts were normalised to the lowest read count in Mothur (n = 10995). Alpha and beta indices were calculated using Mothur and R-script. The Chao and Shannon indices were used as a measurement of alpha-diversity as it takes into account both species richness and the evenness of abundance among the species present in a given sample.

Taxonomic profiles for each metadata group were analysed using the paired Mann-Whitney tests to identify taxa at significantly higher or lower abundance. To determine statistical differences, the Statistical Analysis of Metagenomic Profiles (STAMP) software package was used^43^. The p-values in STAMP was calculated using Welch’s t-test with multiple testing corrections applied using the Bonferroni false discovery rate. Non-metric multidimensional scaling (NMDS) was used to visualise differences in patients with (DS) and without (NoDS) clinical symptoms, using the weighted Unifrac distance matrices (generated by Mothur) in Vegan R package^44^. Adonis (Vegan) was used to calculate significant differences between cohorts and species, using p-value < 0.05.

To test the quality of the samples and the diversity of these samples, we used the Spearman rank correlation in R.

### Quantitative community analysis of bacterial genera and species

After phylotypic assignment to bacterial genera and species, the absolute occurrence (number of sequence reads within subsampled sets) of the OTUs was transformed into relative occurrence for comparison with other samples.

Relative proportions of specific bacterial genera and species, expressed as a percentage of the overall community, were compared within and between each sample site, to determine whether changes in the abundance of specific bacterial genera and/or species could be associated or correlated to the incidence of DS. Relative quantities of bacterial genera present were analysed statistically using a one-way analysis of variance, with a Tukey’s multiple comparisons test, at 95% confidence.

## Supplementary information

Supplementary data

## Data Availability

The datasets generated and/or analysed during the current study are available in the Cardiff University data catalogue, 10.17035/d.2018.0051571814.

## References

[1] Turnbaugh PJ, Gordon JI (2009). The core gut microbiome, energy balance and obesity. The Journal of Physiology.

[2] O’Brien CL, Allison GE, Grimpen F, Pavli P (2013). Impact of colonoscopy bowel preparation on intestinal microbiota. Plos One.

[3] Turnbaugh PJ, Backhed F, Fulton L, Gordon JI (2008). Diet-induced obesity is linked to marked but reversible alterations in the mouse distal gut microbiome. Cell Host and Microbe.

[4] David LA (2013). Diet rapidly and reproducibly alters the human gut microbiome. Nature.

[5] Carmody RN (2015). Diet dominates host genotype in shaping the murine gut microbiota. Cell Host and Microbe.

[6] Marsh PD, Bradshaw DJ (1995). Dental plaque as a biofilm. Journal of Industrial Microbiology.

[7] Jenkinson HF (2011). Beyond the oral microbiome. Environmental Microbiology.

[8] Zarco MF, Vess TJ, Ginsburg GS (2012). The oral microbiome in health and disease and the potential impact on personalized dental medicine. Oral Diseases.

[9] Palmer RJ (2014). Composition and development of oral bacterial communities. Periodontology 2000.

[10] Kilian M, Chapple ILC, Hannig M (2016). The oral microbiome – an update for oral healthcare professionals. British Dental Journal.

[11] Burne RA, Marquis RE (2000). Alkali production by oral bacteria and protection against dental caries. FEMS Microbiology Letters.

[12] Wade WG (2013). The oral microbiome in health and disease. Pharmacological Research: the official journal of the Italian Pharmacological Society.

[13] Buffie CG, Pamer EG (2013). Microbiota-mediated colonization resistance against intestinal pathogens. Nature Reviews. Immunology.

[14] Acharya A (2017). Salivary microbiome in non-oral disease: A summary of evidence and commentary. Archives of Oral Biology.

[15] Dewhirst F (2010). The human oral microbiome. Journal of Bacteriology.

[16] Arendorf TM, Walker DM (1980). The prevalence and intra-oral distribution of *Candida albicans* in man. Archives of Oral Biology.

[17] Newton A. Denture sore mouth: A possible aetiology. *British Dental Journal*. **112**, 357–60 (1962)

[18] Samaranayake LP, Cheung LK, Samaranayake Y (2002). Candidiasis and other fungal diseases of the mouth. Dermatologic Therapy.

[19] Coco BJ (2008). Mixed *Candida albicans* and *Candida glabrata* populations associated with the pathogenesis of denture stomatitis. Oral Microbiology and Immunology [Online].

[20] Dar-Odeh NS, Al-Beyari M, Abu-Hammad OA (2012). The role of antifungal drugs in the management of stomatitis. The International Arabic Journal of Antimicrobial Agents.

[21] Santana IL (2013). Dietary carbohydrates modulate *Candida albicans* biofilm development on the denture surface. PloS ONE.

[22] Cavalcanti YW (2015). Virulence and pathogenicity of *Candida albicans* is enhanced in biofilms containing oral bacteria. Biofouling: Journal of The Bioadhesion and Biofilm Research.

[23] Morse DJ (2018). Denture-associated biofilm infection in three-dimensional oral mucosal tissue models. Journal of Medical Microbiology.

[24] Morse DJ (2019). Modulation of *Candida albicans* virulence in *in vitro* biofilms by oral bacteria. Letters in Applied Microbiology.

[25] O’Donnell LE (2015). The oral microbiome of denture wearers is influenced by levels of natural dentition. Plos ONE.

[26] Shi B (2016). The denture-associated oral microbiome in health and stomatitis. mSphere.

[27] Campos MS, Marchini L, Bernardes LAS, Paulino LC, Nobrega FG (2008). Biofilm microbial communities of denture stomatitis. Oral Microbiology and Immunology.

[28] Kim D (2017). *Candida albicans* stimulates *Streptococcus mutans* microcolony development via cross-kingdom biofilm-derived metabolites. Scientific Reports.

[29] Marsland BJ, Gollwitzer ES (2014). Host-microorganism interactions in lung diseases. Nature Reviews Immunology [Online].

[30] Li Q (2014). Dysbiosis of gut fungal microbiota is associated with mucosal inflammation in Crohn’s disease. Journal of Clinical Gastroenterology.

[31] Takahashi N (2005). Microbial ecosystem in the oral cavity: metabolic diversity in an ecological niche and its relationship with oral diseases. International Congress Series.

[32] Matsubara VH, Bandara HMHN, Mayer MPA, Samaranayake LP (2016). Probiotics as antifungals in mucosal candidiasis. Clinical Infectious Diseases.

[33] Maraki S, Sarchianaki E, Barbagadakis S (2012). *Myroides odoratimimus* soft tissue infection in an immunocompetent child following a pig bite: case report and literature review. Brazilian Journal of Infectious Diseases.

[34] Endicott-Yazdani TR, Dhiman N, Benavides R, Spak CW (2015). *Myroides odoratimimus* bacteremia in a diabetic patient. Proceedings (Baylor University. Medical Center).

[35] Gow NAR (2007). Immune recognition of *Candida albicans* β-glucan by dectin-1. Journal of Infectious Diseases.

[36] Sukhithasri V, Nisha N, Biswas L, Anil Kumar V, Biswas R (2013). Innate immune recognition of microbial cell wall components and microbial strategies to evade such recognitions. Microbiological Research.

[37] McDonald C, Inohara N, Nuñez G (2005). Peptidoglycan signaling in innate immunity and inflammatory disease. Journal of Biological Chemistry.

[38] Schloss PD (2009). Introducing mothur: open-source, platform-independent, community-supported software for describing and comparing microbial communities. Applied and Environmental Microbiology.

[39] Kozich JJ, Westcott SL, Baxter NT, Highlander SK, Schloss PD (2013). Development of a dual-index sequencing strategy and curation pipeline for analyzing amplicon sequence data on the MiSeq Illumina sequencing platform. Appl Environ Microbiol..

[40] Quast C (2013). The SILVA ribosomal RNA gene database project: improved data processing and web-based tools. Nucleic Acids Research..

[41] Wang Q, Garrity GM, Tiedje JM, Cole JR (2007). Naive bayesian classifier for rapid assignment of rRNA sequences into the new bacterial taxonomy. Applied and Environmental Microbiology.

[42] Edgar RC (2010). Search and clustering orders of magnitude faster than BLAST. Bioinformatics.

[43] Parks DH, Beiko RG (2010). Identifying biologically relevant differences between metagenomic communities. Bioinformatics.

[44] Dixon P (2003). (2003). Computer program review VEGAN, a package of R functions for community ecology. Journal of Vegetation Science..

