# Supplementary material for: Molecular community profiling of the bacterial microbiota associated with denture-related stomatitis
Source: Sci Rep. 2019 Jul 15;9:10228. doi: 10.1038/s41598-019-46494-0 (PMC6629705; doi:10.1038/s41598-019-46494-0)
Supplement: Supplementary file 1 — Supplementary data [file 41598_2019_46494_MOESM1_ESM.docx]

Supplementary data

Molecular community profiling of the bacterial microbiota associated with denture-related stomatitis

**Daniel J Morse^1^**, Ann Smith^3^, Melanie J Wilson^2^, Lucy Marsh^3^, Lewis White^4^, Raquel Posso^4^, David J Bradshaw^5^, Xiaoqing Wei^2^, Michael A O Lewis^2^, David W Williams^2^

^1^Microbiomes, Microbes and Informatics Group, School of Biosciences, Cardiff University, Cardiff, UK

^2^Oral and Biomedical Sciences, School of Dentistry, Cardiff University, Cardiff, UK

^3^School of Medicine, Cardiff University, Cardiff, UK

^4^UKCMN Regional Mycology Reference Laboratory, Public Health Wales, Microbiology Cardiff, Cardiff, UK

^5^GlaxoSmithKline Consumer Healthcare, Weybridge, UK

Corresponding author: Daniel J Morse [morsedj@cardiff.ac.uk](mailto:morsedj@cardiff.ac.uk), 07501906510

**Table S1. Top 15 bacterial genera (relative abundance) in samples of palatal mucosa of patients with (DS) and without (NoDS) denture-associated stomatitis, grouped by disease status.**

| **Bacterial Genera** | **Average abundance (%)** | |
| --- | --- | --- |
|  | **DS** | **NoDS** |
| *Streptococcus* | 13.543 | 19.823 |
| *Pseudomonas* | 14.882 | 14.510 |
| *Stenotrophomonas* | 6.302 | 10.913 |
| *Massilia* | 0.008 | 14.118 |
| *Acinetobacter* | 2.627 | 11.160 |
| *Serratia* | 12.126 | 0.001 |
| *Lactobacillus* | 9.977 | 0.065 |
| *Brevundimonas* | 6.700 | 2.800 |
| *Enterococcus* | 0 | 9.087 |
| *Flavobacterium* | 8.435 | 0.006 |
| *Rhizobium* | 4.065 | 0.646 |
| *Chryseobacterium* | 1.822 | 2.712 |
| *Dermacoccus* | 0 | 4.490 |
| *Actinobacillus* | 2.636 | 0.693 |
| *Moraxella* | 3.182 | 0 |
| Other | 13.695 | 8.976 |

**Table S2. Top 15 bacterial genera (relative abundance) in samples of denture-fitting surfaces of patients with (DS) and without (NoDS) denture-associated stomatitis, grouped by disease status.**

| **Bacterial Genera** | **Average abundance (%)** | |
| --- | --- | --- |
|  | **DS** | **NoDS** |
| *Streptococcus* | 19.316 | 35.516 |
| *Actinomyces* | 13.252 | 2.830 |
| *Acinetobacter* | 9.684 | 6.849 |
| *Pseudomonas* | 5.151 | 10.904 |
| *Serratia* | 9.452 | 0.082 |
| *Brevundimonas* | 6.121 | 4.583 |
| *Lactobacillus* | 5.637 | 5.158 |
| *Rhizobium* | 6.964 | 0.318 |
| *Stenotrophomonas* | 0.923 | 8.875 |
| *Myroides* | 0 | 7.220 |
| *Veillonella* | 2.576 | 2.391 |
| *Prevotella* | 3.353 | 1.211 |
| *Microbacterium* | 2.430 | 0.008 |
| *Flavobacterium* | 2.102 | 0 |
| *Haemophilus* | 1.442 | 0.469 |
| Other | 11.597 | 13.586 |

**Table S3. Top 15 bacterial genera (relative abundance) in samples of the tongue of patients with (DS) and without (NoDS) denture-associated stomatitis, grouped by disease status.**

| **Bacterial Genera** | **Average abundance (%)** | |
| --- | --- | --- |
|  | **DS** | **NoDS** |
| *Streptococcus* | 26.156 | 34.499 |
| *Pseudomonas* | 9.607 | 12.798 |
| *Serratia* | 10.364 | 0.007 |
| *Stenotrophomonas* | 3.395 | 6.162 |
| *Brevundimonas* | 3.752 | 5.699 |
| *Enterococcus* | 0.062 | 8.602 |
| *Flavobacterium* | 6.452 | 0.001 |
| *Prevotella* | 4.546 | 1.933 |
| *Actinomyces* | 5.121 | 0.748 |
| *Microbacterium* | 5.371 | 0.022 |
| *Lactobacillus* | 2.714 | 3.257 |
| *Haemophilus* | 3.533 | 1.567 |
| *Granulicatella* | 2.095 | 3.363 |
| *Acinetobacter* | 0.869 | 4.293 |
| *Chryseobacterium* | 1.713 | 2.834 |
| Other | 14.250 | 14.215 |

**Table S4. Top 25 bacterial species (relative abundance) in samples of denture-fitting surfaces of patients with (DS) and without (NoDS) grouped by disease status.**

^a^ indicates unique bacterial species in the top 25 detected bacterial species of either group,

^b^ indicates common bacterial species within the top 25 in both DS and NoDS groups, and other bacterial species may be present in both sample sets but are outside the top 25 detected species.

| **DS** | | **NoDS** | |
| --- | --- | --- | --- |
| **Bacterial species** | **Average abundance (%)** | **Bacterial species** | **Average abundance (%)** |
| *Streptococcus* unclassified | 14.238 | *Serratia liquefaciens* | 13.591 |
| *Myroides* unclassified ^a^ | 8.518 | *Acinetobacter johnsonii* ^b^ | 10.645 |
| *Pseudomonas fluorescens* ^a^ | 7.209 | *Streptococcus mitis* ^b^ | 10.143 |
| *Streptococcus salivarius* ^b^ | 6.786 | *Actinomyces odontolyticus* ^b^ | 9.590 |
| *Brevundimonas terrae* | 6.477 | *Streptococcus salivarius* ^b^ | 6.831 |
| *Pseudomonas putida* ^b^ | 6.180 | *Lactobacillus fermentum* | 6.185 |
| *Agrobacterium tumefaciens* | 4.545 | *Brevundimonas vesicularis* | 4.637 |
| *Acinetobacter lwoffii* ^b^ | 3.933 | *Pseudomonas putida* ^b^ | 3.731 |
| *Acinetobacter johnsonii* ^b^ | 3.733 | *Microbacterium testaceum* ^a^ | 3.481 |
| *Streptococcus mitis* ^b^ | 3.490 | *Veillonella parvula* ^b^ | 3.397 |
| *Chryseobacterium* unclassified | 2.470 | *Streptococcus oralis* ^b^ | 2.680 |
| *Veillonella parvula* ^b^ | 2.411 | *Pseudomonas aeruginosa* | 2.620 |
| *Lactobacillus salivarius* ^b^ | 2.158 | *Acinetobacter lwoffii* ^b^ | 1.915 |
| *Actinomyces lingnae* ^b^ | 1.671 | *Haemophilus parainfluenzae* | 1.716 |
| *Massilia varians* | 1.621 | *Prevotella histicola* | 1.649 |
| *Actinomyces odontolyticus* ^b^ | 1.505 | *Propionibacterium acnes* | 1.457 |
| *Streptococcus oralis* ^b^ | 1.490 | *Streptococcus anginosus* | 1.297 |
| *Lactobacillus rhamnosus* | 1.333 | *Actinomyces lingnae* ^b^ | 0.986 |
| *Streptococcus sobrinus* ^a^ | 1.264 | *Streptococcus sanguinis* | 0.796 |
| *Devosia* unclassified | 1.222 | *Prevotella melaninogenica* | 0.790 |
| *Lactobacillus paracasei* | 1.186 | *Granulicatella adiacens* | 0.775 |
| *Stenotrophomonas nitritireducens* | 1.006 | *Leptotrichia unclassified* ^a^ | 0.668 |
| *Acinetobacter junii* | 0.953 | *Lactobacillus salivarius* ^b^ | 0.658 |
| *Delftia tsuruhatensis* ^a^ | 0.947 | *Actinomyces graevenitzii* | 0.630 |
| *Empedobacter brevis* ^a^ | 0.734 | *Lactobacillus unclassified* | 0.623 |
| **Total** | **91.929** | **Total** | **84.884** |

**Table S5. Top 25 bacterial species (relative abundance) in samples of palatal mucosa of patients with (DS) and without (NoDS) denture-associated stomatitis grouped by disease status.**

^a^ indicates unique bacterial species in the top 25 detected bacterial species of either group,

^b^ indicates common bacterial species within the top 25 in both DS and NoDS groups, and other bacterial species may be present in both sample sets but are outside the top 25 detected species.

| **DS** | | **NoDS** | |
| --- | --- | --- | --- |
| **Bacterial species** | **Average abundance (%)** | **Bacterial species** | **Average abundance (%)** |
| *Pseudomonas fluorescens* ^b^ | 19.992 | *Serratia liquefaciens* | 16.698 |
| *Massilia aurea* ^a^ | 15.572 | *Pseudomonas putida* | 16.583 |
| *Streptococcus* unclassified | 15.338 | *Lactobacillus paracasei* | 13.869 |
| *Enterococcus faecalis* ^a^ | 12.754 | *Brevundimonas vesicularis* ^b^ | 6.516 |
| *Dermacoccus nishinomiyaensis* ^a^ | 6.297 | *Stenotrophomonas nitritireducens* ^a^ | 4.532 |
| *Streptococcus oralis* ^b^ | 4.016 | *Moraxella osloensis* ^a^ | 4.477 |
| *Acinetobacter johnsonii* ^b^ | 3.839 | *Agrobacterium tumefaciens* ^a^ | 3.611 |
| *Chryseobacterium* unclassified ^b^ | 3.819 | *Stenotrophomonas* unclassified ^a^ | 3.366 |
| *Massilia varians* ^a^ | 3.334 | *Acinetobacter johnsonii* ^b^ | 3.182 |
| *Brevundimonas terrae* ^b^ | 2.646 | *Streptococcus salivarius* | 2.794 |
| *beta proteobacterium* ^a^ | 2.252 | *Phormidium species* | 2.781 |
| *Sphingomonas* species ^a^ | 1.576 | *Streptococcus mitis* | 2.759 |
| *Devosia* unclassified ^a^ | 1.541 | *Streptococcus oralis* ^b^ | 2.562 |
| *Brevundimonas vesicularis* ^b^ | 1.016 | *Pseudomonas fluorescens* ^b^ | 2.344 |
| *Massilia* unclassified ^a^ | 0.732 | *Chryseobacterium scophthalmum* ^a^ | 2.014 |
| *Rhizobium* unclassified ^b^ | 0.712 | *Propionibacterium acnes* | 1.914 |
| *Acidovorax* unclassified | 0.664 | *Luteimonas cucumeris* ^a^ | 1.411 |
| *Propionibacterium acnes* | 0.393 | *Stenotrophomonas maltophilia* | 0.836 |
| *Veillonella parvula* | 0.232 | *Streptococcus sanguinis* ^b^ | 0.639 |
| *Delftia tsuruhatensis* | 0.229 | *Chryseobacterium* unclassified ^b^ | 0.551 |
| *Streptococcus sanguinis* ^b^ | 0.223 | *Brevundimonas terrae* ^b^ | 0.498 |
| *Brevundimonas* unclassified | 0.201 | *Rhizobium* unclassified ^b^ | 0.443 |
| *Rhizobium mesosinicum* ^a^ | 0.198 | *Streptococcus infantis* | 0.420 |
| *Massilia alkalitolerans* ^a^ | 0.198 | *Pseudoxanthomonas mexicana* | 0.413 |
| *Methylobacterium* unclassified ^a^ | 0.186 | *Acinetobacter lwoffii* ^a^ | 0.322 |
| **Total** | **97.959** | **Total** | **95.531** |

**Table S6. Top 25 bacterial species (relative abundance) in samples of the tongue of patients with (DS) and without (NoDS) grouped by disease status.**

^a^ indicates unique bacterial species in the top 25 detected bacterial species of either group,

^b^ indicates common bacterial species within the top 25 in both DS and NoDS groups, and other bacterial species may be present in both sample sets but are outside the top 25 detected species.

| **DS** | | **NoDS** | |
| --- | --- | --- | --- |
| **Bacterial species** | **Average abundance (%)** | **Bacterial species** | **Average abundance (%)** |
| *Streptococcus salivarius* ^b^ | 18.412 | *Streptococcus salivarius* ^b^ | 15.372 |
| *Pseudomonas fluorescens* ^b^ | 12.481 | *Serratia liquefaciens* | 11.432 |
| *Enterococcus faecalis* | 11.527 | *Streptococcus mitis* ^b^ | 11.185 |
| *Streptococcus* unclassified | 8.806 | *Pseudomonas putida* | 5.940 |
| *Streptococcus oralis* ^b^ | 6.500 | *Microbacterium maritypicum* ^a^ | 5.205 |
| *Streptococcus mitis* ^b^ | 6.091 | *Streptococcus oralis* ^b^ | 4.725 |
| *Brevundimonas terrae* | 4.958 | *Actinomyces odontolyticus* | 4.017 |
| *Granulicatella adiacens* | 4.199 | *Acidovorax* unclassified | 3.828 |
| *Chryseobacterium* unclassified | 3.814 | *Stenotrophomonas nitritireducens* ^a^ | 3.311 |
| *Lactobacillus rhamnosus* | 3.709 | *Lactobacillus paracasei* | 2.978 |
| *Methylophilus methylotrophus* ^a^ | 2.873 | *Pseudomonas aeruginosa* ^a^ | 2.315 |
| *Brevundimonas vesicularis* ^b^ | 2.224 | *Haemophilus parainfluenzae* ^b^ | 2.167 |
| *Acinetobacter johnsonii* ^b^ | 2.017 | *Pseudomonas fluorescens* ^b^ | 2.060 |
| *Rothia mucilaginosa* ^b^ | 1.688 | *Brevundimonas vesicularis* ^b^ | 2.033 |
| *Prevotella tannerae* | 0.853 | *Propionibacterium acnes* | 1.637 |
| *Streptococcus sanguinis* | 0.754 | *Citrobacter freundii* | 1.635 |
| *Devosia* unclassified ^a^ | 0.730 | *Granulicatella adiacens* | 1.605 |
| *Propionibacterium acnes* | 0.635 | *Chryseobacterium scophthalmum* ^a^ | 1.464 |
| *Pseudoxanthomonas mexicana* ^a^ | 0.634 | *Veillonella parvula* | 0.843 |
| *Haemophilus parainfluenzae* ^b^ | 0.575 | *Afipia broomeae* ^a^ | 0.796 |
| *Lactobacillus plantarum* | 0.515 | *Microbacterium testaceum* ^a^ | 0.782 |
| *Prevotella oris* | 0.509 | *Actinomyces lingnae* | 0.753 |
| *Dermacoccus nishinomiyaensis* | 0.405 | *Acinetobacter johnsonii* ^b^ | 0.729 |
| *Streptococcus sobrinus* ^a^ | 0.379 | *Rothia mucilaginosa* ^b^ | 0.705 |
| *Veillonella parvula* | 0.370 | *Prevotella melaninogenica* ^a^ | 0.637 |
| **Total** | **95.656** | **Total** | **88.153** |

**Table S7. Primer sequences for *Candida* detection of clinical samples by nested-PCR.**

| **Primer/probe name (target)** | **Sequence (5’ 🡪 3’)** | **Melting temp. (°C)** |
| --- | --- | --- |
| L18R | GCC-TGC-TTT-GAA-CAC-TCT | 54.0 |
| L18F | CTC-GTA-GTT-GAA-CCT-TGG | 54.0 |
| Pan-*Candida* | ATC-TTT-TTG-ATG-CGT-ACT-GGA-CCC-TG | 52.9 |
| *C. glabrata* | GGC-TAA-CCC-CAA-GTC-CTT-GTG-GCT-T | 55.9 |
| *C. krusei* | TAC-CTA-TGG-TAA-GCA-CTG-TTG-CGG-C | 54.2 |
